# MicroRNA-320c inhibits tumorous behaviors of bladder cancer by targeting Cyclin-dependent kinase 6

**DOI:** 10.1186/s13046-014-0069-6

**Published:** 2014-09-02

**Authors:** Xiao Wang, Jian Wu, Yiwei Lin, Yi Zhu, Xianglai Xu, Xin Xu, Zhen Liang, Shiqi Li, Zhenghui Hu, Xiangyi Zheng, Liping Xie

**Affiliations:** 1 Department of Urology, The First Affiliated Hospital, School of Medicine, Zhejiang University, 79 Qingchun Road, Hangzhou 310003, Zhejiang Province, People¿s Republic of China

**Keywords:** miR-320c, CDK6, Bladder cancer, Proliferation, Migration, Invasion

## Abstract

**Background:**

Increasing evidence has suggested that dysregulation of microRNAs (miRNAs) could contribute to human disease including cancer. Previous miRNA microarray analysis illustrated that miR-320c is down-regulated in various cancers. However, the roles of miR-320c in human bladder cancer have not been well elucidated. Therefore, this study was performed to investigate the biological functions and molecular mechanisms of miR-320c in human bladder cancer cell lines, discussing whether it could be a therapeutic biomarker of bladder cancer in the future.

**Methods:**

Two human bladder cancer cell lines and samples from thirteen patients with bladder cancer were analyzed for the expression of miR-320c by quantitative RT-PCR. Over-expression of miR-320c was established by transfecting mimics into T24 and UM-UC-3. Cell proliferation and cell cycle were assessed by cell viability assay, flow cytometry and colony formation assay. Cell motility ability was evaluated by transwell assay. The target gene of miR-320c was determined by luciferase assay, quantitative RT-PCR and western blot. The regulation of cell cycle and mobility by miR-320c was analyzed by western blot.

**Results:**

We observed that miR-320c was down-regulated in human bladder cancer tissues and bladder cancer cell lines T24 and UM-UC-3. Over-expression of miR-320c could induce G1 phase arrest in UM-UC-3 and T24 cells, and subsequently inhibited cell growth. We also indentified miR-320c could impair UM-UC-3 and T24 cell motility. In addition, we identified CDK6, a cell cycle regulator, as a novel target of miR-320c. Moreover, we demonstrated miR-320c could induce bladder cancer cell cycle arrest and mobility via regulating CDK6. We also observed that inhibition of miR-320c or restoration of CDK6 in miR-320c-over-expressed bladder cancer cells partly reversed the suppressive effects of miR-320c.

**Conclusions:**

miR-320c could inhibit the proliferation, migration and invasion of bladder cancer cells via regulating CDK6. Our study revealed that miR-320c could be a therapeutic biomarker of bladder cancer in the future.

## Background

Urinary bladder cancer is generally accepted as the 11th most commonly diagnosed type of cancer worldwide [1]. In the US, statistics illustrated that an estimated 74,690 cases were newly diagnosed bladder cancer, among which 15,580 were expected to die in 2014 [2]. Although it is believed that both environmental [3] and genetic factors [4],[5], such as genetic polymorphism, chromosomal anomalies and epigenetic changes, play critical roles in the development of bladder cancer, the exact mechanisms of bladder carcinogenesis are still not well elucidated. Therefore, understanding the potential carcinogenetic mechanisms of these genetic changes is important to identify novel therapeutic targets and prognostic biomarkers.

MicroRNAs (miRNAs) are small (20?~?23 nucleotides), endogenous, non-coding RNAs, which constitute a novel cluster of target gene regulators [6]. They are involved in various cellular processes, including self-renewal, proliferation, metabolism and apoptosis, by inducing post-transcriptional gene repression via accelerating the degradation and/or blocking the translation of their target mRNAs [7]. The miRNA genes were observed to be specifically deleted in leukemia initially illustrated the important role of miRNA in carcinogenesis [8]. Subsequent researches have demonstrated that the expression of specific miRNAs is altered in many types of cancer, which is associated with carcinogenesis and cancer progression [9]¿[13]. Meanwhile, accumulating evidences illustrated that the development and progression of bladder cancer is closely related to the aberrant expression of miRNAs [14]. The initial study of miRNA expression in bladder cancer was reported by Gottardo in 2007 and 10 up-regulated miRNAs were detected [15].

Previous miRNA microarray analysis illustrated that miR-320 is down-regulated in breast cancer, acute myelogenous leukemia and colon cancer, revealing that miR-320 could probably act as a tumor suppressor in prohibiting the behavior of cancer [16]¿[18]. It was reported that miR-320 could inhibit prostate cancer cell proliferation by down-regulating the Wnt/beta-catenin signaling pathway [19]. Additionally, miR-320a/c/d could inhibit the migration and invasion of hepatocellular cancer via targeting GNAI1, a crucial protein of multiple cellular signal transduction pathways [20]. Moreover, Iwagami et al. showed that miR-320c regulated the resistance of pancreatic cancer cells to gemcitabine via SMARCC1 (a core subunit of the switch/sucrose nonfermentable), suggesting that miR-320c could be a potential therapeutic target in pancreatic cancer [21]. Nevertheless, the potential mechanism of miR-320c in bladder cancer has not been well elucidated.

In our present study, we further testified miR-320c expression pattern in bladder cancer tissue. Additionally, for the first time, we detected that miR-320c could suppress growth and motility of the human bladder cancer cell line T24 and UM-UC-3. The tumor inhibitive role and potential mechanisms of miR-320c on bladder cancer were determined.

## Methods

### Reagents

The miR-320c mimic (named as miR-320c) and the negative control duplex (named as NC) lacking any significant homology to all known human sequences were used for transient gain of function research. For colony formation assay, the 2?-O-Methyl modified duplexes of both miR-320c and NC were used. 2?-O-Methyl modified miR-320c inhibitor (named as miR-320c-Inh) and NC inhibitor (named as Inh-NC) were used for observing the reversed effect of over-expression of miR-320c. The small interference RNA targeting human CDK6 mRNA (named as siCDK6) was synthesized as described previously [22], which targeted nucleotides 1424¿1442 according to Genbank accession NM_001145306.1. All RNA duplexes were chemically synthesized by GenePharma Corporation (Shanghai, China). All the applied sequences were listed in Table 1.

**Table 1:** The oligonucleotides used in this study

**Name**^**a**^	**Sequence (5?-?>?3?)**
miR-320c mimics (sense)	AAAAGCUGGGUUGAGAGGGU
NC (sense)	ACUACUGAGUGACAGUAGA
miR-320c inhibitor	ACCCUCUCAACCCAGCUUUU
microRNA inhibitor NC	CAGUACUUUUGUGUAGUACAA
siCDK6 (sense)	CUGGAAAGGUGCAAAGAAAdTdT
miR-320c F	AAAAGCTGGGTTGAGAGGGT
U6 F	TGCGGGTGCTCGCTTCGGCAGC
CDK6 F	GGATAAAGTTCCAGAGCCTGGAG
CDK6 R	GCGATGCACTACTCGGTGTGAA
GAPDH F	AAGGTGAAGGTCGGAGTCA
GAPDH R	GGAAGATGGTGATGGGATTT
CDK6-Wt F	cAATCAATGCAAGAGTGATTG*CAGCTTTA*TGTTCATTTGTTTGTTTGTTg
CDK6-Wt R	tcgacAACAAACAAACAAATGAACA*TAAAGCTGC*AATCACTCTTGCATTGATTgagct
CDK6-Mut F	cAATCAATGCAAGAGTGATTGgtcgaaatTGTTCATTTGTTTGTTTGTTg
CDK6-Mut R	tcgacAACAAACAAACAAATGAACAatttcgacCAATCACTCTTGCATTGATTgagct

^a^F, forward primer; R, reverse primer.

### Tissue samples

Paired bladder cancer tissues and para-carcinoma bladder mucosal tissues were acquired from patients receiving radical cystectomy. The samples were gained between Jan 2011 and June 2011 from the First Affiliated Hospital, School of Medicine, Zhejiang University (Hangzhou, P.R. China) with informed consent and Ethics Committee¿s approval. The clinical data of the patients were listed in Table 2. All tissue samples were stored in liquid nitrogen before use.

**Table 2:** Clinical data of the patients

**Patient no.**	**Sex**	**Age**	**TNM stage**	**Histological grade**
1	M	62	T2N0M0	III
2	M	60	T1N0M0	I
3	M	53	T1N0M0	III
4	M	86	T1N0M0	III
5	M	55	T1N0M0	II
6	F	74	T2N0M0	III
7	M	56	T2N0M0	III
8	F	76	T3N0M0	III
9	M	65	T2N0M0	II
10	F	69	T2N0M0	II
11	M	72	T3N0M0	III
12	M	78	T1N0M0	II
13	M	76	T3N0M0	III

### Cell culture and transfection

The human bladder cancer cell lines UM-UC-3, T24, and non-tumor urothelial cell line SV-HUC-1 (Shanghai Institute of Cell Biology, Chinese Academy of Sciences) were cultured in RPMI1640 medium (Gibco) containing 10% heat-inactivated fetal bovine serum (Gibco), 50U/ml penicillin and 50 ?g/ml streptomycin under a humid atmosphere including 5% CO2 at 37°C. Cells were plated to 60¿70% confluency in medium without antibiotics 1 day before transfection. Lipofectamine 2000 Reagent (Invitrogen, Carlsbad, CA, USA) was selected for transfection under the guide of the instruction.

### RNA isolation and real-time PCR

Expression level of miR-320c and CDK6 in tissues and cell lines was calculated by quantitative real-time RT-PCR. Small RNA was extracted from both frozen samples and cell lines with RNAiso Kit for Small RNA (TaKaRa, Japan) and subsequently reverse transcribed into cDNA with One Step PrimeScript miRNA cDNA Synthesis Kit (TaKaRa, Japan). Meanwhile, total RNA from cell lines UM-UC-3, T24, and SV-HUC-1 was extracted using RNAiso plus (TaKaRa, Japan) and transcribed into cDNA using PrimeScript RT reagent Kit (TaKaRa, Japan). The resulting cDNA of miR-320c and CDK6 was quantified by SYBR Premix Ex Taq (TaKaRa, Japan) via an ABI 7500 fast real-time PCR System (Applied Biosystems, Carlsbad, USA). Moreover, the cycle threshold (Ct) value was used for our analysis (?Ct), and we determined the expression of small nuclear RNA U6 and GAPDH mRNA as internal controls to calculate the relative expression levels of miR-320c and CDK6 via the 2^-??Ct^ (delta-delta-Ct algorithm) method. All the primers were listed in Table 1.

### Cell viability assay

Each well of 96-well plate was plated with 4000 cells (UM-UC-3 or T24). After 24 h incubation, the cells were transfected with RNA duplexes (25¿100nM). After 48 h incubation, medium in each well was removed before cell counting solution (WST-8, Dojindo Laboratories, Tokyo, Japan) was added to it and incubated for another 2 h. The absorbance of the solution was measured spectrophotometrically at 450 nm with MRX II absorbance reader (Dynex Technologies, Chantilly, VA, USA).

### Colony formation assay

UM-UC-3 and T24 cells were incubated for 24 h after transfected with 2?-O-Methyl modified duplexes (50nM). Five hundreds of transfected cells were seeded in a new six-well plate and cultivated continuously for another 10 days. Cells were subsequently treated with methanol and 0.1% crystal violet for fixing and staining. The colony formation rate was calculated via the following equation: colony formation rate?=?(number of colonies/number of seeded cells)?×?100%.

### Cell migration and invasion assay

The 24-well Boyden chamber with 8 ?m pore size polycarbonate membrane (Corning, NY) was used for evaluating the cell motility. Matrigel was used to pre-coat the membrane to simulate a matrix barrier for invasion assay. Four thousands of cells were seeded on the upper chamber with 200 ?l serum-free medium after transfected with RNA duplex for 48 h. 600 ?l medium with 20% serum, served as a chemoattractant, was added to the lower chamber. After 24 h incubation, the membranes were fixed with methanol and stained with 0.1% crystal violet. Five visual fields (×200) were randomly selected from each membrane, and the cell numbers were counted via a light microscope.

### Cell cycle analysis by flow cytometry

After 48 h transfection, UM-UC-3 and T24 cells were washed with PBS and fixed in 75% ethanol at ?20°C. After 24 h fixation, the cells were washed with PBS and treated with DNA Prep Stain (Beckman Coulter, Fullerton, CA) for 30 min. Cell cycle analysis was conducted by BD LSRII Flow Cytometry System with FACSDiva software (BD Bioscience, Franklin Lakes, USA). The cell cycle distribution was illustrated as the percentage of cells in G1, S, and G2 populations and data was evaluated by ModFit LT software package.

### Protein extraction and Western blotting analysis

After 48 h transfection with RNA duplexes, UM-UC-3 and T24 cells were lysed in cell lysis buffer and concentration of total protein in every lysate was quantified using the BCA Protein Assay kit (Pierce). Equivalent amounts (30¿50 ?g) of protein were separated by 10% SDS-polyacrylamide gels and transferred to polyvinylidene difluoride membranes. Membranes were blocked for 1 h with 5% non-fat milk and then incubated at 4°C overnight with specific primary antibody at appropriate dilutions according to the instructions. After washed three times in TBS-Tween, the membranes were incubated with the corresponding horseradish peroxidase (HRP)-conjugated secondary antibody for 1 h and detected by an enhanced chemi-luminescence (ECL) system (Pierce Biotechnology Inc., Rockford, IL). The primary immunoblotting antibodies used were: anti-GAPDH, anti-CDK6 (Epitomics, Burlingame, CA).

### Luciferase assays

In order to construct the luciferase reporter vectors, the 3?-UTR (untranslated region) of CDK6 was designed (Sangon, Shanghai, China), which contained putative target region for miR-320c (sequence set in Table 1). The synthesized oligonucleotide pair was annealed at 90°C for 3 min and then transferred to 37°C for another 15 min to form a duplex before inserted into pmirGLO Dual-Luciferase miRNA Target Expression Vector (Promega, USA) between the SacI and SalI sites. Additionally, the mutant miR-320c putative target region was also designed, annealed and inserted into pmirGLO Dual-Luciferase Vector in the same way (sequence set in Table 1). Both insertions were verified by sequencing (Sangon, Shanghai, China).

HEK 293 T cells were cultivated in a 24-well plate for 24 h before co-transfected with 50nM of either miR-320c mimic or NC oligos and 200 ng reporter plasmid containing wild type (Wt) or mutant type (Mut) of CDK6 3?-UTR. After 48 h transfection, the relative luciferase activity was calculated by Dual-Luciferase Reporter Assay System (Promega, USA).

### miR-320c inhibitor experiments

To further verify the function of miR-320c, the antisense inhibitor (miR-320c inhibitor) experiments were performed to see whether the reverse effects to over-expression could be observed. The cells were co-transfected with either miR-320c mimics or NC oligos with miR-320c inhibitor or NC inhibitor [23]. After 48 h of transfection, colony formation assay, flow cytometry and transwell assay (cell migration and invasion assay) was used to analyze the cell proliferation, cell cycle and cell motility. Besides, expression level of miR-320c and CDK6 was calculated by quantitative real-time RT-PCR. In addition, the CDK6 expression was further determined by Western blotting.

### CDKrescue experiments

The pTarget-CDK6 plasmid was constructed via inserting the human CDK6 coding sequence without the 3?-UTR into the pTarget vector (GeneCopoeia, USA), and verified by sequencing. The T24 cells were co-transfected with either miR-320c mimics or NC oligos with pTarget-CDK6 (pCDK6) or empty pTarget vector (pNull). After 48 h of transfection, colony formation assay, flow cytometry and transwell assay was used to evaluate the cell proliferation, cell cycle and cell motility. Additionally, the CDK6 expression was determined by Western blotting.

### Statistical analysis

All the statistics were expressed as mean?±?standard deviation (SD) of three independent experiments. GraphPad Prism version 5 for Windows was used to conduct all the relative analyses via either the student¿s *t*-test or Two-way ANOVA. P?<?0.05 was considered to be statistically significant.

## Results

### miR-320c is down-regulated in bladder cancer

**Figure 1:**
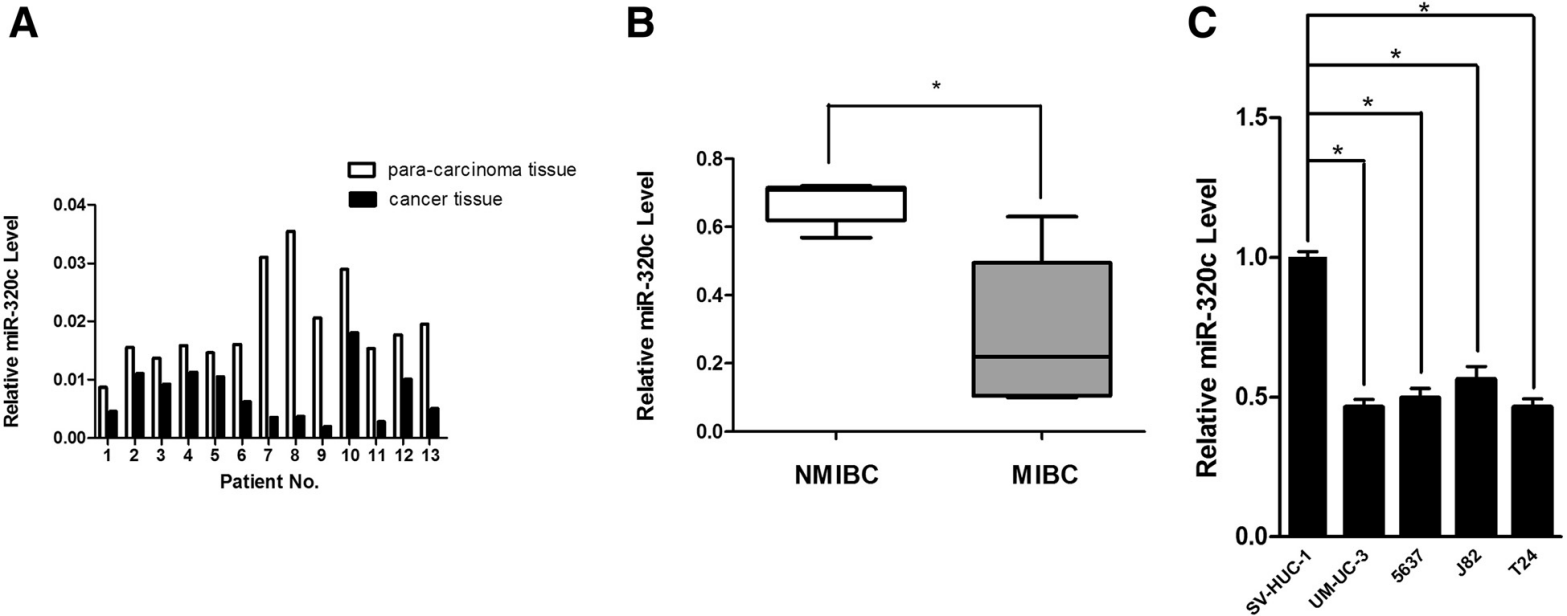
**miR-320c is down-regulated in bladder cancer Expression levels for miR-320c by real-time PCR analysis were normalized with U6. (A)** Individual expression value of miR-320c for both cancer and matched normal tissues (calculated by 2^-?Ct^). **(B)** The relationship between NMIBC and MIBC was shown in a box and whiskers graph. Box-plot lines represented medians and interquartile ranges of the normalized threshold values, and whiskers indicated 10¿90th percentiles. The expression level of miR-320c was significantly lower in MIBC compared with NMIBC. **(C)** The miR-320c levels in 4 bladder cancer cell lines were lower compared with SV-HUC-1 cell line.

The expression pattern of miR-320c in human bladder cancer has not been analyzed. Therefore, we used real-time RT-PCR to quantify the expression levels of miR-320c in 13 pairs of human bladder cancer tissues and adjacent normal mucosal tissues. Compared with their non-cancerous counterparts, it was observed that miR-320c expression levels were lower in cancerous tissues, and 6 out of 13 samples illustrated a 50% reduction (Figure 1A). We also illustrated the expression value for both cancer and matched normal tissues for miR-320c normalized to U6 RNA in Table 3. In addition, we compared the expression pattern of miR-320c between muscle invasive bladder cancer (MIBC) and non muscle invasive bladder cancer (NMIBC), and we found the expression of miR-320c was lower in MIBC compared to NMIBC, which indicated that low level of miR-320c could be associated with tumor aggressiveness and poor prognosis (Figure 1B). However, such relationship should be further verified in a larger sample set in the future. Furthermore, 4 bladder cancer cell lines (UM-UC-3, T24, 5637, J82) demonstrated similar expression pattern of miR-320c compared with non-tumor urothelial cell line SV-HUC-1 (Figure 1C). Therefore, it was speculated that miR-320c could be a potential tumor suppressor in bladder cancer.

**Table 3:** Expression value of miR-320c in cancer and matched normal tissues (normalized by U6 RNA)

	**Cancer tissues (2**^**-?Ct**^**)**	**Normal tissues (2**^**-?Ct**^**)**	**Folds (2**^**-??Ct**^**)**
1	0.004581387	0.008668512	0.53
2	0.011048543	0.015517070	0.71
3	0.009226505	0.013696964	0.67
4	0.011280697	0.015843117	0.71
5	0.010525262	0.014578640	0.72
6	0.006258358	0.016064279	0.39
7	0.003569654	0.031034140	0.12
8	0.003721242	0.035402621	0.10
9	0.002008035	0.020617311	0.10
10	0.018073253	0.028955877	0.63
11	0.002800694	0.015303442	0.18
12	0.010096506	0.017701311	0.57
13	0.005083367	0.019505165	0.26

### miR-320c suppresses bladder cancer cell viability, inhibits clone formation and triggers G1-phase arrest

**Figure 2:**
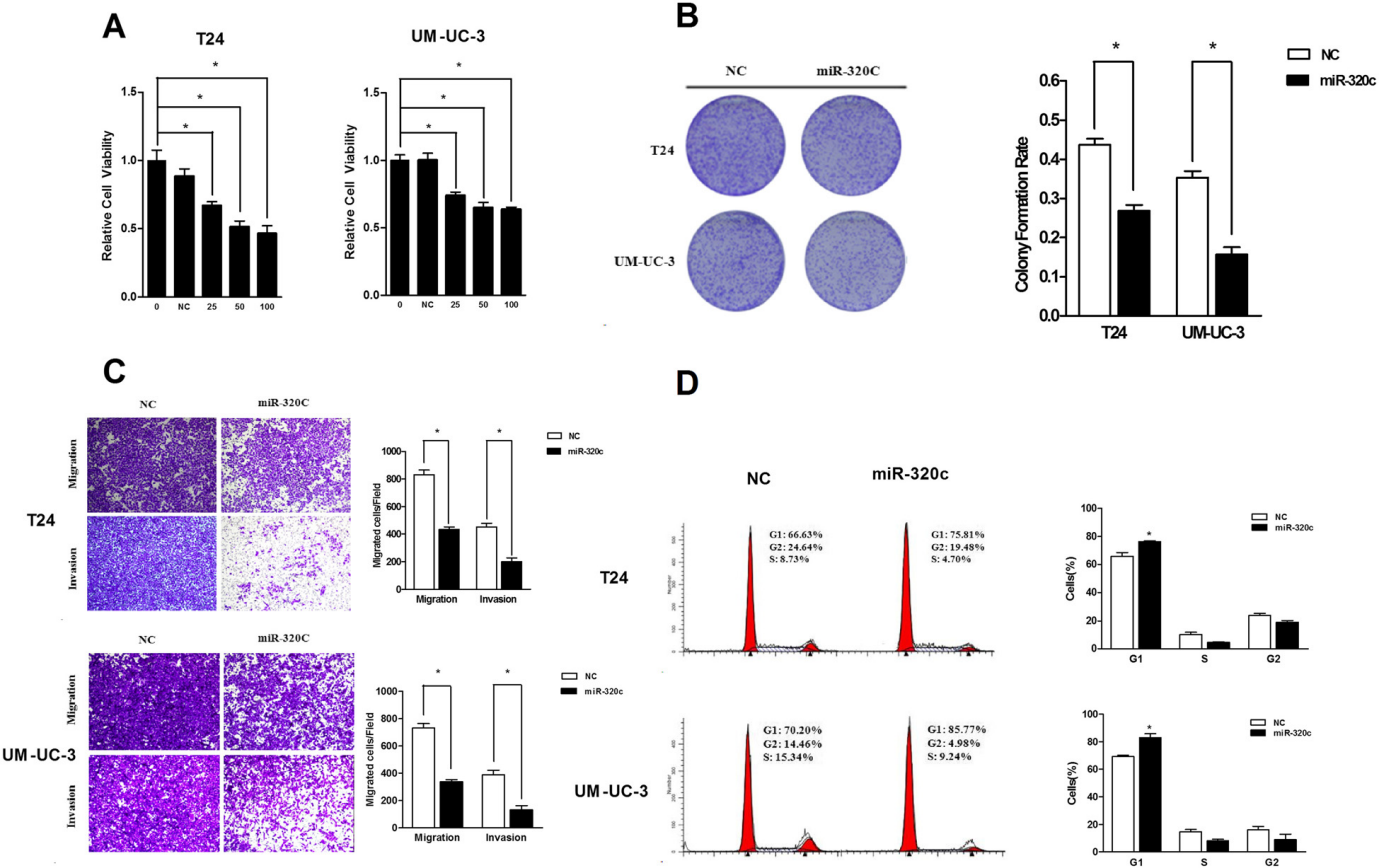
**Over-expression of miR-320c suppresses bladder cancer cell proliferation and motility. (A)** Cell viability assay. The relative cell viability was lower in the miR-320c treated groups (cell viability of 0nM was regarded as 1.0), respectively. **(B)** Colony formation assay (representative wells were presented). The colony formation rate was lower in miR-320c treated groups. **(C)** miR-320c impaired the motility of both cell lines (representative migration and invasion results at?×?200 were presented). **(D)** Cell cycle distribution in bladder cancer cell lines. Over-expression of miR-320c induced G1-phase arrest in both cell lines (representative histograms were presented) (*P?<?0.05).

In order to understand the potential mechanisms of miR-320c in tumor suppressing, the bladder cancer cell lines were transfected with miR-320c to evaluate the effect of over-expression via cell viability assay. As a result, miR-320c illustrated a significant inhibitory effect on bladder cancer cell viability in a dose-dependent manner (Figure 2A). After 48 h transfection, miR-320c (50nM) could reduce cell viability in both UM-UC-3 and T24 cell by 35% and 49%, respectively. Furthermore, miR-320c potently inhibited the colony forming ability in both cell lines. Compared with cell lines transfected with NC, the colony formation rate decreased drastically in those transfected with miR-320c (Figure 2B).

Additionally, in order to better clarify the underlying mechanisms for miR-320c inhibiting cancer cell proliferation, we transfected the cells with 50nM miR-320c 48 h before assessing the impact of miR-320c on cell cycle distribution via flow cytometry. As a result, we observed a significant increase in the percentage of cells in the G1/G0 phase and a decrease in the percentage of cells in the S and G2/M phase in miR-320c-overexpressing cells (Figure 2D). These results suggested that miR-320c could lead to G1-phase arrest.

### miR-320c impairs UM-UC-3 and T24 cell motility

To further elucidate the function of miR-320c, we investigated the potential effect of miR-320c on UM-UC-3 and T24 cell motility. As illustrated by the transwell assay, over-expression of miR-320c decreased the migration and invasion of cancer cells compared with NC (Figure 2C). Therefore, miR-320c negatively regulated the motility of UM-UC-3 and T24 cells.

### CDK6 is a key regulator in miR-320c mediated cell proliferation suppression, cell cycle arrest and cell motility impairment

**Figure 3:**
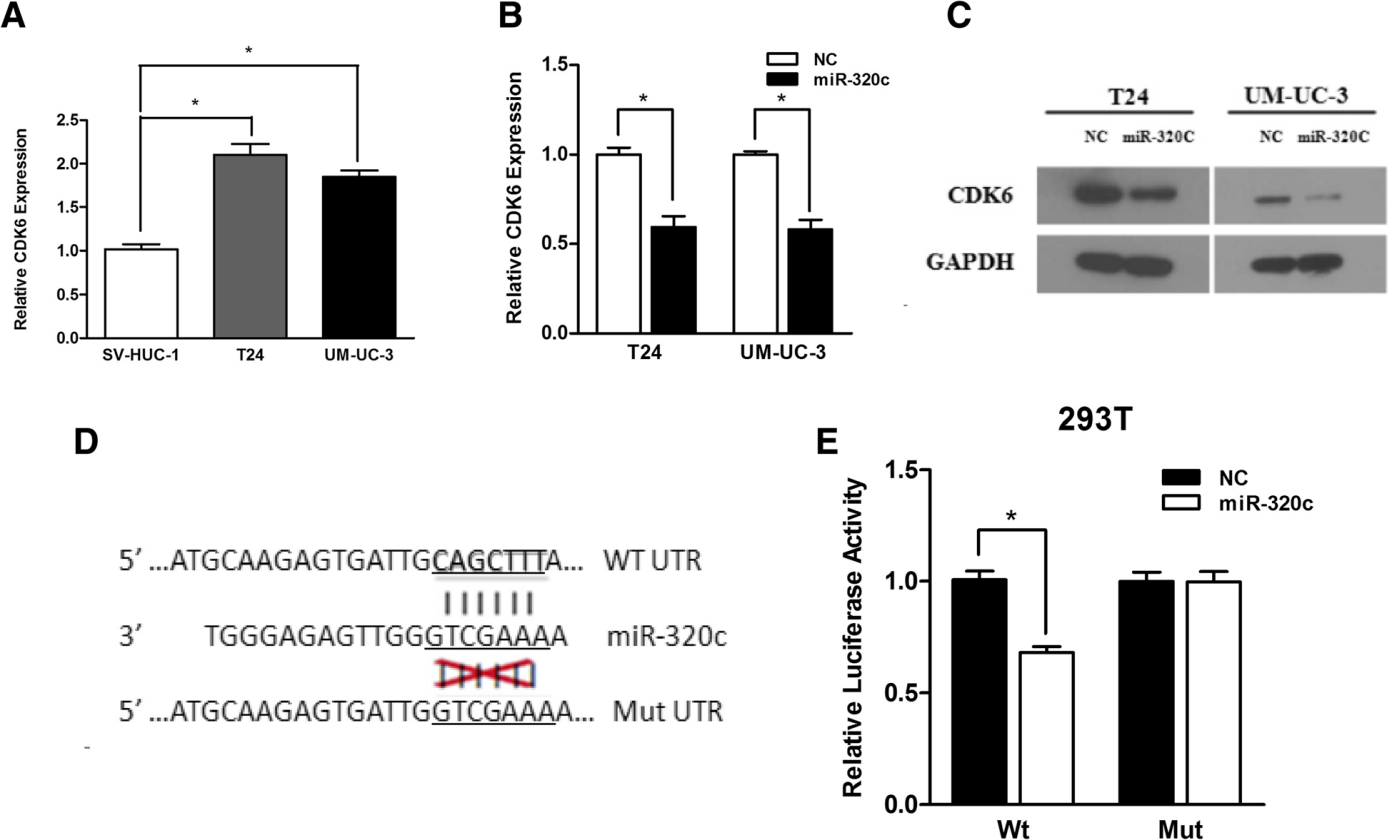
**CDK6 is a direct target of miR-320c. (A)** An increased expression pattern of CDK6 was observed in UM-UC-3 and T24 cells compared with SV-HUC-1 cells. **(B, C)** Over-expression of miR-320c reduced CDK6 expression level in both cell lines significantly (levels of mRNA and protein). **(D)** A predicted seed region in the 3?-UTR of CDK6 was illustratred (top). The mutated sequence was highlighted in underline (bottom). **(E)** 293 T cells were co-transfected with 50nM of either miR-320c mimic or NC oligos and 200 ng plasmid containing Wt or Mut of CDK6 3¿-UTR. The relative firefly luciferase activity normalized with Renilla luciferase was calculated 48 h after transfection (*P?<?0.05).

We used TargetScan analysis (http://www.targetscan.org), miRTarBase (http://mirtarbase.mbc.nctu.edu.tw) and MicroCosm Targets (http://www.ebi.ac.uk/enright-srv/microcosm/htdocs/targets/v5/) to detect the potential downstream targets of miR-320c. Among all the candidate genes predicted by the online tools, CDK6, a potential downstream target of miR-320c, was of particular interest because all online tools indicated that it had a very high scoring predicted binding site and CDK6 was considered to be a positive cell cycle regulator (G1/S transition) in many types of cancer [24]¿[26]. Additionally, we also searched for information on conservation of CDK6 among species. The NCBI database illustrates that CDK6 gene is conserved in many species, including chimpanzee, dog, cow, mouse, rat, zebra fish, fruit fly, mosquito and C.elegans (http://www.ncbi.nlm.nih.gov/homologene/963). Previous study indicated that the expression of CDK6 increased drastically in bladder cancerous tissues compared with their non-cancerous counterparts and elevated CDK6 expression resulted in the development of bladder cancer [26]. In our study, an increased expression pattern of CDK6 was observed in the human bladder cancer cell lines UM-UC-3 and T24 compared with non-tumor urothelial cell line SV-HUC-1 (Figure 3A). Moreover, we verified that the expression of CDK6 drastically reduced in both levels of mRNA and protein after the transfection of miR-320c, which was consistent with the cell cycle arrest phenomenon (Figure 3B, C).

### CDK6 is a novel direct target of miR-320c

In order to clarify whether CDK6 was a direct downstream target of miR-320c, the synthesized 3?-UTR of CDK 6 was cloned into down-stream of firefly luciferase of pmirGLO Dual-Luciferase miRNA Target Expression Vector. Additionally, we also constructed another vector with mutated putative binding sites (Figure 3D). The results illustrated that HEK 293 T cells transiently transfected with the Wt-3?- UTR-reporter and miR-320c exhibited drastically reduced relative luciferase activity compared with co-transfection of Wt and NC. However, co-transfection of Mut CDK6 3?-UTR and miR-320c or NC did not affect the relative luciferase activity (Figure 3E). Therefore, CDK6 was considered to be a direct downstream target of miR-320c based on the luciferase assays.

### Inhibition of miR-320c partially reverses the over-expression of miR-320c induced effects

**Figure 4:**
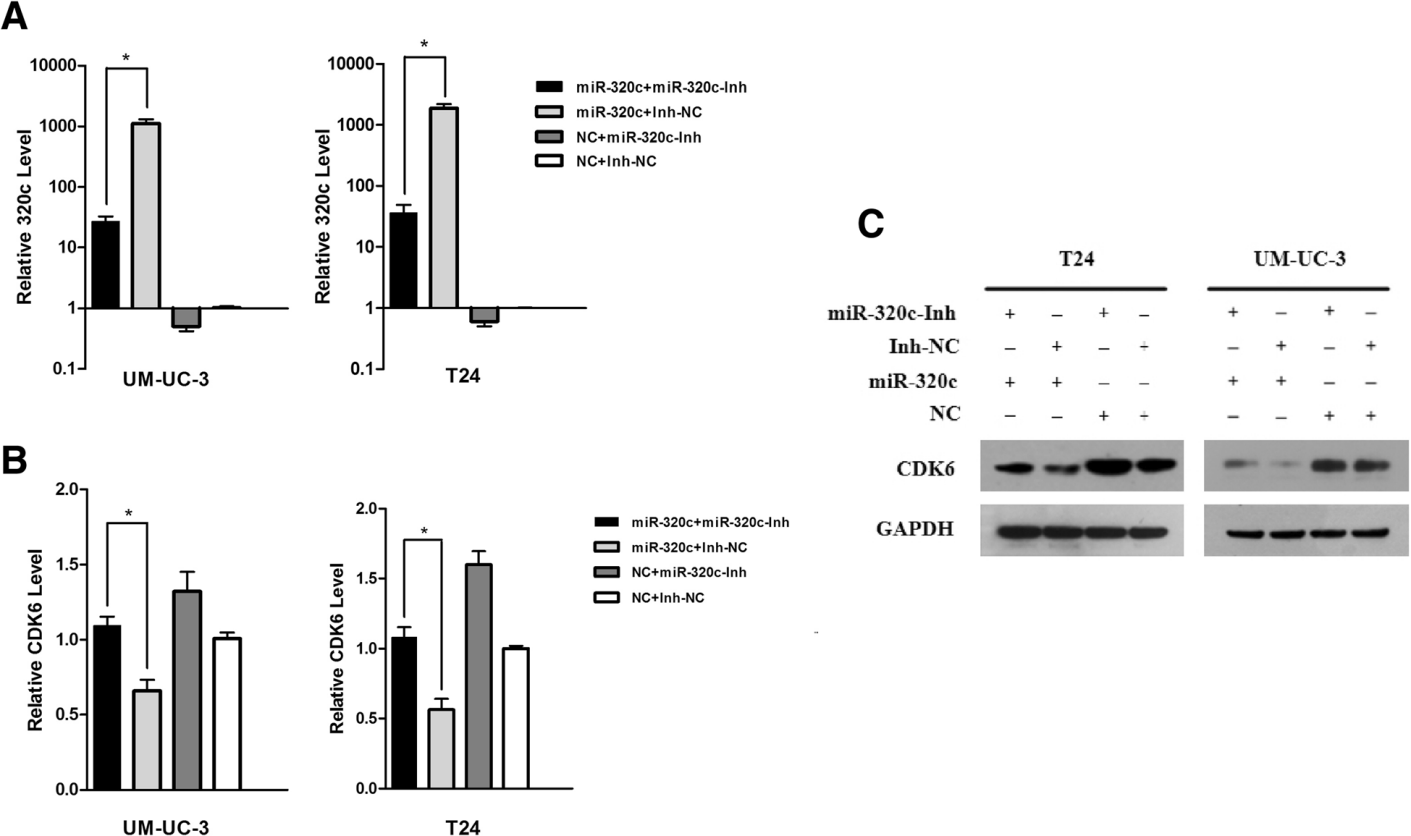
**Ectopic miR-320c expression and inhibition of miR-320c suppress the expression of miR-320c and CDK6.** T24 and UM-UC-3 cells were co-transfected with miR-320c-Inh (vs. Inh-NC) and miR-320c (vs. NC). **(A)** The expression of miR-320c was determined by real-time PCR. **(B,C)** The expression of CDK6 was determined by real-time PCR and western blot analysis. GAPDH served as an internal control (*P?<?0.05).

**Figure 5:**
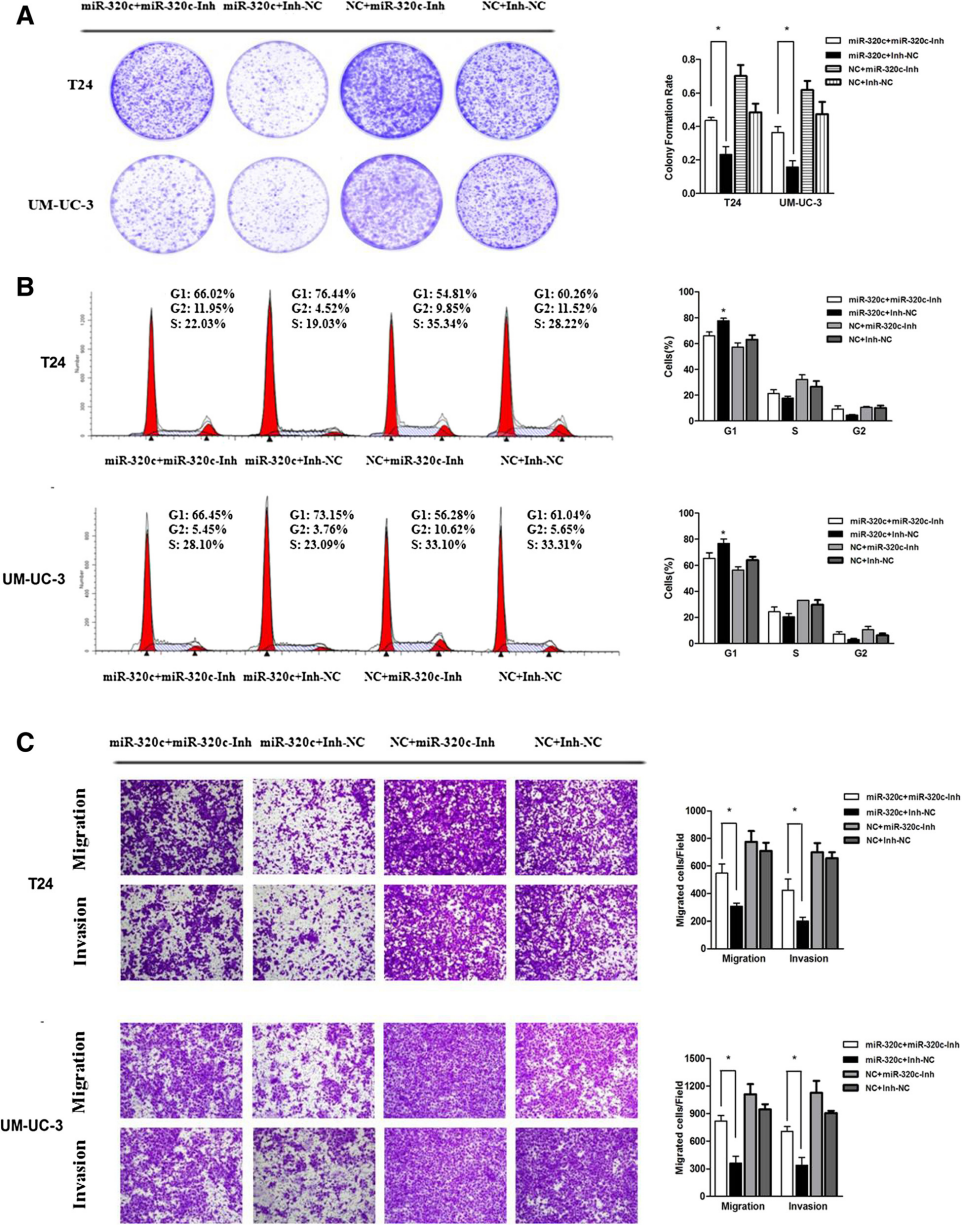
**Inhibition of miR-320c partially reverses the over-expression of miR-320c induced effect. (A, B)** Co-transfection of miR-320c-Inh could partially attenuate the effect of miR-320c on the colony formation rate and cell cycle arrest in the T24 and UM-UC-3 cell lines. **(C)** The bladder cancer cells migration and invasion ability was restored after miR-320c-Inh transfection (×200) (*P?<?0.05).

To better verify the function of miR-320c, the antisense inhibitor (miR-320c inhibitor) experiments were performed to see whether the reverse effects to over-expression could be observed. As a result, co-transfection of miR-320c-Inh was applied to attenuate the miR-320c expression promotion and the CDK6 expression inhibition by miR-320c in the level of mRNA and protein (Figure 4A-C). Furthermore, miR-320c-Inh could partially reverse the effect of miR-320c on cell proliferation inhibition and cell cycle arrest in the T24 and UM-UC-3 cell lines (Figure 5A,B). A significant decrease in the percentage of cells in the G1/G0 phase and an increase in the G2/M phase was observed, which indicating that transfection of miR-320c-Inh could attenuate the G1-phase arrest by miR-320c. Additionally, the bladder cancer cells migration and invasion ability was restored after miR-320c-Inh transfection (Figure 5C). Thus, we confirmed that miR-320c-Inh could reverse the effects to over-expression of miR-320c.

### Repression of CDK6 plays essential roles in miR-320c-induced bladder cancer inhibition effect

**Figure 6:**
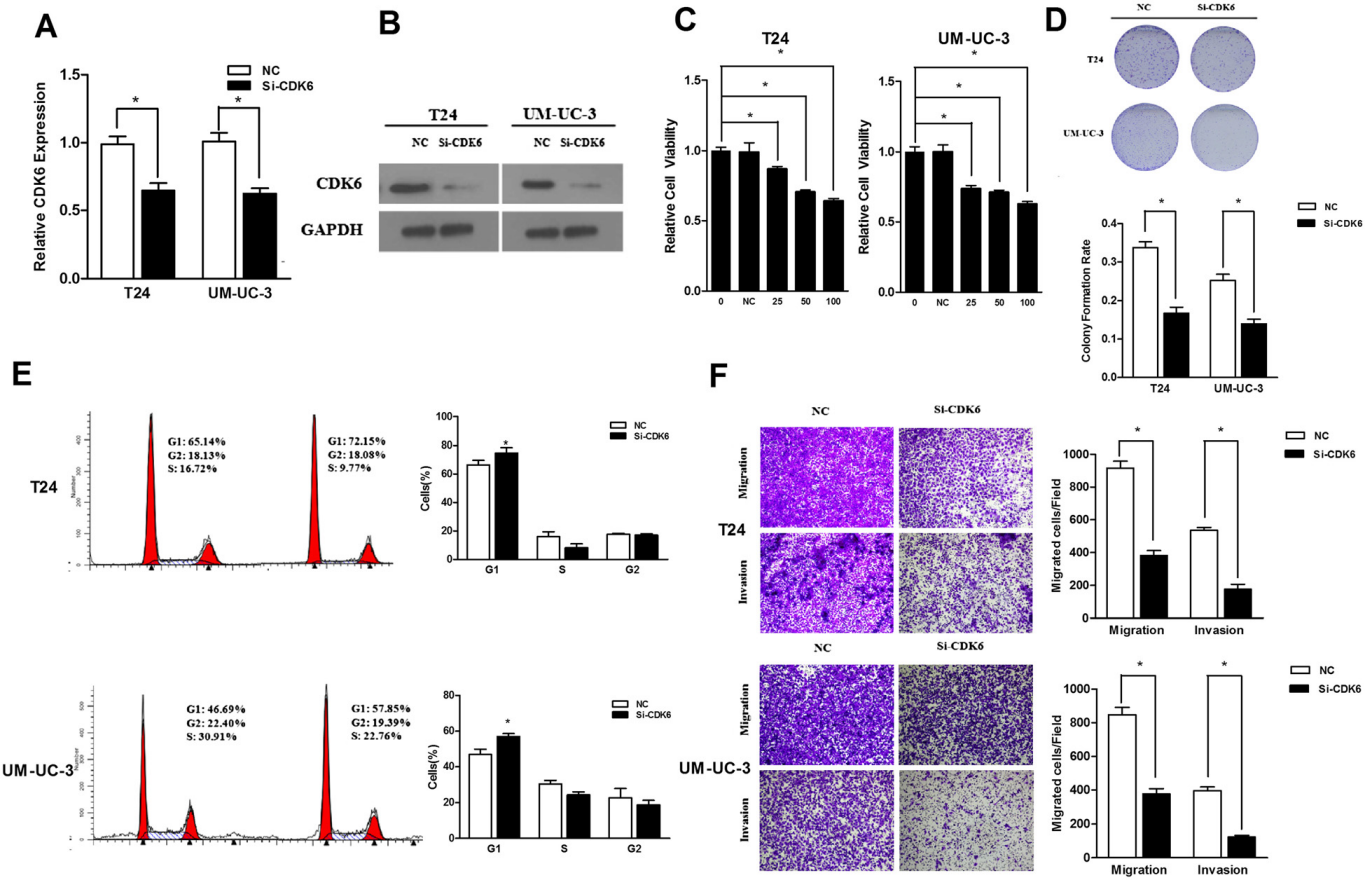
**Knock-down of CDK6 phenocopied the effect of miR-320c. (A, B)** The knock-down of CDK6 via RNAi technique reduced the expression of CDK6 (levels of mRNA and protein). **(C)** SiCDK6 suppressed bladder cancer cell growth. **(D)** SiCDK6 reduced the colony formation rate in both cell lines (representative wells were presented). **(E)** SiCDK6 induced G1-phase arrest in both cell lines (representative histograms were presented). **(F)** SiCDK6 yield an inhibitory effect on invasion and migration in both cell lines (×200) (*P?<?0.05).

Furthermore, we used loss of function approach to evaluate whether the physiological function of CDK6 was involved in miR-320c regulated cancer inhibition effect. The knock-down of CDK6 via RNAi technique dramatically decreased the expression of CDK6 in mRNA and protein levels in both cell lines (Figure 6A,B). Moreover, the transfection of siCDK6 significantly suppressed the proliferation of bladder cancer cell lines, and we also observed a significant increase in the percentage of cells in the G1/G0 phase and a decrease in the S and G2/M phase, which phenocopied the effects of miR-320c on bladder cancer cells (Figure 6C-E). Interestingly, the knock-down of CDK6, generally accepted as a cell cycle mediator, also yield an inhibitory effect on cell invasion and migration (Figure 6F). Therefore, we further verified that miR-320c inhibited tumorous behaviors of bladder cancer cells by targeting CDK6.

### Restoration of CDK6 expression partially rescues miR-320c-induced suppression of tumorous behavior

**Figure 7:**
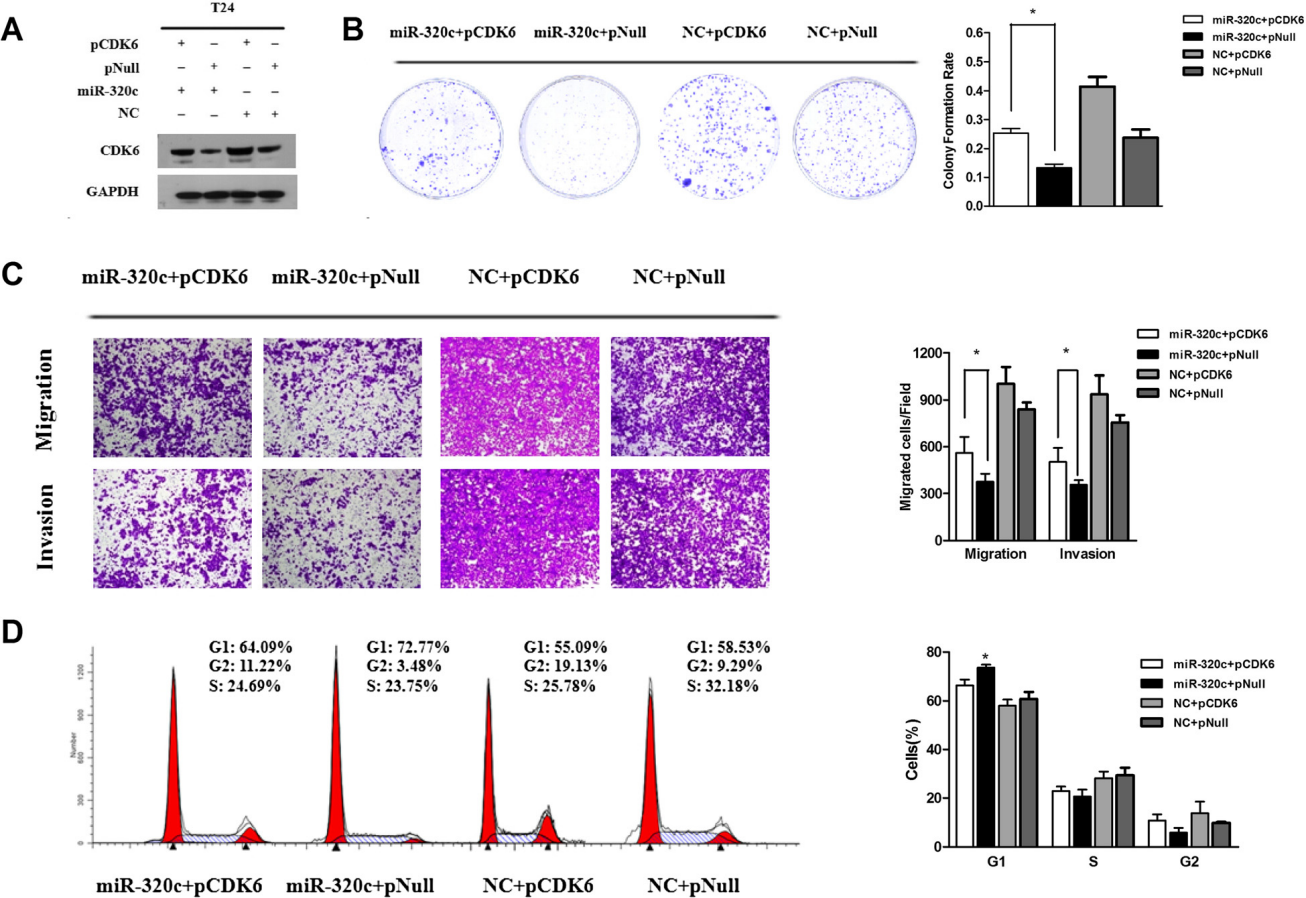
**Forced expression of CDK6 partly rescued miR-320c-dependent suppression of tumorous behavior.** The T24 cells were co-transfected with either miR-320c mimics or NC oligos with pTarget-CDK6 or empty pTarget vector. **(A)** The expression of CDK6 or GAPDH was detected by Western blot analysis. **(B)** Forced CDK6 expression partly attenuated the inhibitory effect of miR-320c on the colony formation rate. **(C)** Co-transfection of pCDK6 partially rescued miR-320c-induced inhibitory effect on cell invasion and migration (×200). **(D)** Forced expression of CDK6 significantly abrogated cell cycle arrest effect of miR-320c (*P?<?0.05).

We had verified that over-expression of miR-320c could induce G1-phase arrest, suppression of cell invasion and migration before and we wondered whether forced CDK6 expression could abrogate the cell cycle arrest and promote cell motility by miR-320c. In parallel, co-transfection of pCDK6 was applied to attenuate the CDK6 expression inhibition by miR-320c (Figure 7A). Forced CDK6 expression partially, but significantly, promoted cell proliferation and motility (Figure 7B, C). We also observed a significant decrease in the percentage of cells in the G1/G0 phase and an increase in the G2/M phase, which indicating that co-transfection of pCDK6 and miR-320c could attenuate the G1-phase arrest by miR-320c (Figure 7D). Thus, we confirmed that CDK6 was a key mediator of tumor suppression function of miR-320c in bladder cancer.

## Discussion

During the past decades, effective targeted therapies of bladder cancer contributing to improved prognosis were the highlight of researches [27]. In recent years, a growing number of researches illustrated that abnormal expression of miRNAs was considered to be a key regulator in carcinogenesis [28],[29]. Moreover, aberrant expression profiles of miRNA in cancer detected by microarray analysis provided deeper insights into the molecular passages of carcinogenesis [17],[18],[30]. A previous systematic review summarized the dysfunction of miRNAs in bladder cancer, which would help to establish a mature system in diagnosis and therapy using miRNAs in the future [14]. However, limited studies were focused on the regulative functional role of miRNAs in bladder cancer. The impact of specific miRNAs in bladder was still poorly understood. Thereafter, our institution performed some researches to elucidate the potential relationship between bladder cancer and miRNAs [31],[32].

To the best of our knowledge, we initially detected a decreased expression pattern of miR-320c in human bladder cancer tissue compared with its normal adjacent tissue in the study, Recent miRNA microarray analyses demonstrated that miR-320 was down-regulated in many types of cancer, including breast cancer, acute myelogenous leukemia and colon cancer, indicating that miR-320 could act as a tumor suppressor in cancer, which was similar to our results [16]¿[18]. Furthermore, previous studies also revealed that miR-320c could inhibit the motility of hepatocellular cancer and regulate the resistance of pancreatic cancer cells to gemcitabine [20],[21]. However, owing to unique genetic background in different types of cancer, the biological function of miR-320c in bladder cancer was not well elucidated. Therefore, this is the first study to determine the functional role of miR-320c in bladder cancer. Considering both of our tissue samples and cell lines are from patients with muscle-invasive bladder cancer, the outcome of this study is probably more meaningful in muscle-invasive or recurrent cancer.

Our study illustrated that miR-320c was down-regulated in bladder cancer tissues compared with normal adjacent tissues, though the sample size was relatively small. Similar result was detected in 4 bladder cancer cell lines compared with non-tumor urothelial cell line SV-HUC-1, which further strengthened the conclusion that miR-320c was down-regulated in bladder cancer. A gain-of- function study was further conducted in bladder cancer cell lines. When both UM-UC-3 and T24 cells were transfected with miR-320c, we observed that miR-320c could suppress bladder cancer cell viability and inhibit clone formation. In addition, flow cytometry indicated that miR-320c could trigger G1-phase arrest, which could be the potential mechanism of miR-320c-regulated proliferation inhibition. Moreover, cell motility assay demonstrated that over-expression of miR-320c impaired bladder cancer cells migration and invasion ability.

To elucidate the possible mechanism responsible for the anticancer behaviors triggered by miR-320c, we conducted a computerized analysis for the potential target. Therefore, we identified CDK6 as a new target of miR-320. A previous study illustrated that CDK6 was over-expressed in bladder cancer tissue [26]. In our present study, similar expression pattern of CDK6 was observed in the human bladder cancer cell lines, which suggested the oncogenic role of CDK6 in bladder cancer. PCR and Western blot study indicated that miR-320c could dramatically inhibit CDK6 expression and luciferase assay further confirmed that CDK6 was a downstream target of miR-320c via directly binding to the 3?-UTR.

To better verify the function of miR-320c, the antisense inhibitor (miR-320c inhibitor) experiments were performed. We confirmed that miR-320c-Inh could reverse the effects to over-expression of miR-320c. miR-320c-Inh could partially reverse the effect of miR-320c on cell cycle arrest and suppressing cell proliferation and motility.

As a critical cell cycle regulator, CDK6 induces an important cascade of events in G1-phase. It can modify Rb phosphorylation efficiently together with CDK4 and cyclin D1, and is considered to a primary sensor for driving cells through the R point to enter a new round of replication. Therefore, CDK6 has been regarded as a possible target for cancer therapy [33]. The knock-down of CDK6 via RNAi technique illustrated the G1-phase arrest, which phenocopied the cell cycle arrest effect of miR-320c over-expression.

Therefore, CDK6 is another important mediator in miR-320c induced G1/S phase transition arrest and cell proliferation suppression.

As we mentioned before, the knock-down of CDK6, generally accepted as a cell cycle mediator, also yielded an inhibitory effect on cell mobility, which was confusing. Previous studies also indicated that knock-down of CDK6 could inhibit cell invasion and migration in gastric and Ewing¿s Sarcoma [34]. However, the accurate mechanisms were still unknown. A recent study indicated that CDK6, as a key protein, coordinated cell proliferation and migration in breast cancer mainly dependent on the expression of estrogen receptor [35]. Furthermore, various oncogenic signaling pathways, including c-Myc, Ras, and Neu (ErbB2), have been described to converge on cell cycle proteins cyclinD1, CDK4/6 expression [36]. The data presented in our study also identified a novel role for cell cycle protein CDK6 in bladder cancer through the coordination of cell cycle, migration and invasion.

Ectopic over-expression of CDK6 (without the 3?-UTR) significantly abrogated the miR-320c-induced G1 arrest of bladder cancer cells and promoted cell proliferation and motility in vitro. To sum up, these results suggested that miR-320c inhibited the proliferation and motility of bladder cancer cells via, at least in part, directly targeting the 3?-UTR of CDK6. Thus, our current study revealed what we believed to be a novel upstream regulatory mechanism of CDK6 in cancer cells.

## Conclusions

In conclusion, our study suggests that miR-320c is a potential tumor suppressor in bladder cancer. By targeting CDK6, miR-320c can inhibit proliferation and impair cell mobility in bladder cancer cells. Restoration of miR-320c could be a promising therapeutic strategy for bladder cancer therapy.

## Abbreviations

miRNA: MicroRNA

miR-320c: MicroRNA-320c

CDK6: Cyclin-dependent kinase 6

qPCR: Quantitative RT-PCR assays

WT: Wild type

Mut: Mutant type

## Competing interests

All authors declare that they have no competing interests.

## Authors¿ contributions

XW, YWL, ZL and SQL performed and participated in analysis of laboratory experiments data. XW, JW and LPX participated in the design of experiments. XW, XXL, XX and YZ acquired, preserved clinical samples. YWL, XYZ and LPX provided administrative support and funded experiments. XW, JW and ZHH drafted the manuscript. All authors have contributed and approved the final manuscript.
